# Practical implications of tumor proximity to landmark vessels in minimally invasive radical antegrade modular pancreatosplenectomy

**DOI:** 10.1007/s13304-023-01584-7

**Published:** 2023-07-17

**Authors:** Emanuele Federico Kauffmann, Niccolò Napoli, Armando Di Dato, Alice Salamone, Michael Ginesini, Cesare Gianfaldoni, Virginia Viti, Gabriella Amorese, Carla Cappelli, Fabio Vistoli, Ugo Boggi

**Affiliations:** 1 Division of General and Transplant Surgery, University of Pisa, Azienda Ospedaliero Universitaria Pisana, Via Paradisa 2, 56124 Pisa, Italy; 2 Division of Anesthesia and Intensive Care, Azienda Ospedaliero Universitaria Pisana, Pisa, Italy; 3 Diagnostic and Interventional Radiology, Azienda Ospedaliero Universitaria Pisana, Pisa, Italy

**Keywords:** Robotic, Laparoscopic, Minimally-invasive, Pancreatectomy, Distal pancreatectomy, RAMPS

## Abstract

Careful preoperative planning is key in minimally invasive radical antegrade modular pancreatosplenectomy (MI-RAMPS). This retrospective study aims to show the practical implications of computed tomography distance between the right margin of the tumor and either the left margin of the spleno-mesenteric confluence (d-SMC) or the gastroduodenal artery (d-GDA). Between January 2011 and June 2022, 48 minimally invasive RAMPS were performed for either pancreatic cancer or malignant intraductal mucinous papillary neoplasms. Two procedures were converted to open surgery (4.3%). Mean tumor size was 31.1 ± 14.7 mm. Mean d-SMC was 21.5 ± 18.5 mm. Mean d-GDA was 41.2 ± 23.2 mm. A vein resection was performed in 10 patients (20.8%) and the pancreatic neck could not be divided by an endoscopic stapler in 19 operations (43.1%). In patients requiring a vein resection, mean d-SMC was 10 mm (1.5–15.5) compared to 18 mm (10–37) in those without vein resection (*p* = 0.01). The cut-off of d-SMC to perform a vein resection was 17 mm (AUC 0.75). Mean d-GDA was 26 mm (19–39) mm when an endoscopic stapler could not be used to divide the pancreas, and 46 mm (30–65) when the neck of the pancreas was stapled (*p* = 0.01). The cut-off of d-GDA to safely pass an endoscopic stapler behind the neck of the pancreas was 43 mm (AUC 0.75). Computed tomography d-SMC and d-GDA are key measurements when planning for MI-RAMPS.

## Introduction

Pending the results of the international multicenter prospective randomized DIPLOMA trial [1], the Miami international evidence-based guidelines recommend that minimally invasive distal pancreatectomy (MI-DP) for pancreatic cancer is feasible, safe, and oncologically equivalent to the open technique in experienced hands [2].

Despite the lack of high-level evidence about sound oncologic advantage [3], either open (RAMPS) or minimally-invasive radical antegrade modular pancreatosplenectomy (MI-RAMPS) are frequently used for resection of left-sided pancreatic cancer to improve lymph node yields and increase the rate of radical resection [4]. Compared to open surgery, a minimally invasive approach reduces time to functional recovery and improves the quality of life [5], making MI-RAMPS an appealing alternative to open surgery. However, MI-RAMPS adds technical complexity over conventional MI-DP [6, 7].

Anticipation of technical difficulty is key in all minimally invasive procedures. For MI-DP several scores are available [6–9]. Tumor proximity to major vessels, typically defined as a distance ≤ 2 cm between the medial margin of the tumor and the root of the splenic artery and/or the splenomesenteric confluence, is a well-recognized difficulty factor in MI-DP [6–9]. However, implications of tumor proximity to major vessels in terms of either need for vein resection or challenges in the transection of the neck of the pancreas have not been assessed specifically.

The aim of this study is to define the practical implications of the distance between the right margin of the tumor and either the left margin of the spleno-mesenteric confluence (d-SMC) or the gastroduodenal artery (d-GDA) with respect to the need for vein resection and the possibility to use a stapler to divide the neck of the pancreas, respectively.

## Methods

A prospectively maintained institutional database was retrospectively analyzed to identify MI-RAMPS for pancreatic cancer and malignant intraductal papillary mucinous neoplasms performed between January 2011 and June 2022.

This study was performed according to the principles of the Declaration of Helsinki [10]. Surgery was performed either laparoscopically or robotically (da Vinci Surgical System, Intuitive Surgical, Sunnyvale, CA, USA) based on the availability of the robotic system. For robotic procedures either a daVinci Si or Xi System was used.

### Study design

This is an intention-to-treat study aiming to retrospectively define the implications of d-SMC and d-GDA with respect to the possibility of vein resection and the ability to use and endoscopic stapler to divide the neck of the pancreas in MI-RAMPS, respectively.

### Patient selection

At our Institution, RAMPS is electively performed in all patients with left-sided pancreatic tumors associated with a high probability of invasion of the extrapancreatic nerve plexus (i.e. pancreatic cancer and malignant intraductal mucinous papillary neoplasms). Indication of RAMPS was established by a multidisciplinary tumor board. Eligibility for MI-RAMPS was based upon the anticipated tolerability of pneumoperitoneum and the possibility to achieve radical tumor resection. Patients with overt tumor abutment to the superior mesenteric-portal vein (SM-PV) were excluded. Involvement of the celiac trunk, following primary chemotherapy, was not considered an absolute contraindication to MI-RAMPS. There were no specific selection criteria with respect to laparoscopic or robotic RAMPS. However, robotic assistance was preferred when the robot was timely available.

### Radiology measurements

**Fig. 1:**
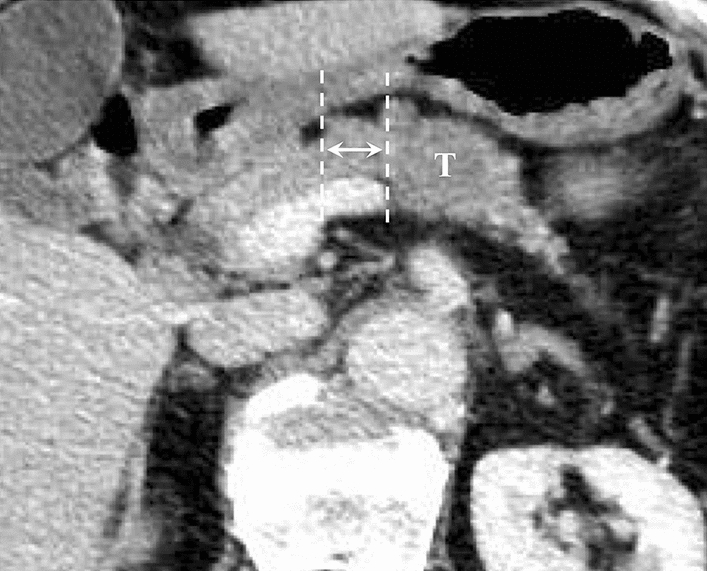
Arrow between vertical dotted lines indicates d-SMC. T = tumor

**Fig. 2:**
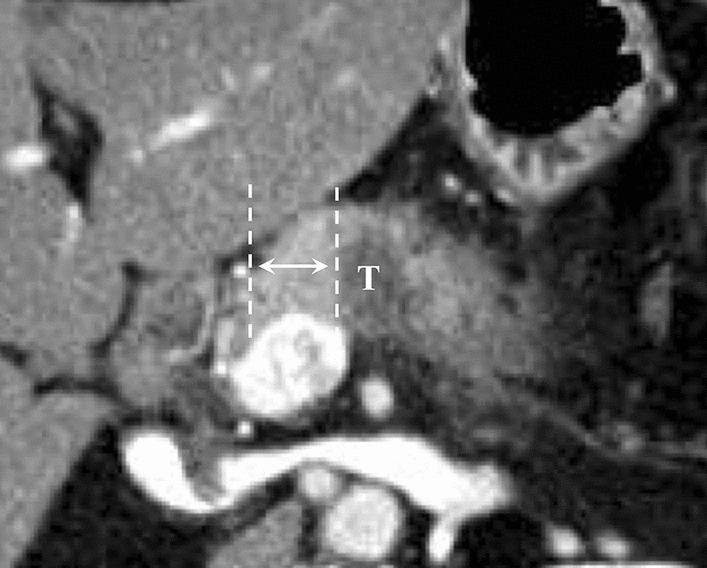
Arrow between vertical dotted lines indicates d-GDA. T = tumor

All patients had a high-quality contrast-enhanced computed tomography (CT) scan within 4 weeks of surgery. All CT scans were reviewed by an expert radiologist (CC). Besides all information required for tumor diagnosis and staging, the radiologist defined both d-SMC and d-GDA in millimeters (Figs. 1 and 2). Measurements were taken between the outmost right-sided tumor margin and target vessels. In patients who had received neoadjuvant FOLFIRINOX chemotherapy, the outmost right-sided area of “fibrosis” was considered.

### Outcome data

The following outcome variables were reported: need for vein resection, use of a stapler for division of the neck of the pancreas, operative time, conversion to open surgery, estimated blood loss, need for intraoperative blood transfusions, incidence and severity of postoperative complications, clinically relevant postoperative pancreatic fistula (grade B and C postoperative pancreatic fistula), post-pancreatectomy hemorrhage, delayed gastric emptying, chyle leak, length of hospital stay, 30-day readmission, post-operative mortality, tumor histology and stage, margin status, number of examined lymph nodes, and histology of resected vascular segments.

Operative time was calculated from induction of pneumoperitoneum to skin closure.

Conversion to open surgery was defined as any additional incision required to complete the operation, excluding the one used for specimen extraction. Conversions occurring in hemodynamically stable patients were considered elective conversions. Conversions occurring in hemodynamically unstable patients, typically due to difficult-to-control hemorrhage, were considered emergency conversions [11].

Estimated blood loss calculated by the difference between the amount of fluids in the suction canister and the volume of fluids used for irrigation [12].

Postoperative complications were graded according to Clavien-Dindo. Complications graded ≥ 3 were considered severe [13]. The overall burden of postoperative complications was estimated for each patient using the comprehensive complication index (CCI) [14]. Pancreas-specific complications were defined and graded according to the International Study Group on Pancreatic Surgery [15–18]. Post-operative mortality was defined as any death occurring during the first 90 days or in-hospital if the initial hospital stay was longer than 90 days.

Specimens were analyzed as previously described [19], and the 1 mm rule (i.e. tumor cells ≤ 1 mm of any margin) was used to define microscopic margin positivity (R1) [20]. The AJCC-TNM 8^th^ edition staging system was employed.

### Surgical technique

The technique of robotic RAMPS was previously described, including details on vein resection and reconstruction [21]. The same principles are applied to laparoscopic RAMPS. Vein resections were classified according to the International Study Group on Pancreatic Surgery [22].

We recently reported also on alternative options for division and closure of the pancreatic neck [23]. Briefly, when a tunnel cannot be safely developed between the neck of the pancreas and the SM-PV, and when a stapler cannot be safely employed, the neck of the pancreas is transected using harmonic shears. The pancreatic duct is selectively identified and ligated, and the parenchyma is closed with interrupted sutures.

### Statistical analysis

Categorical variables are presented as rates and proportions. Continuous variables are reported as mean ± SD if normally distributed or as the median and interquartile range (IQR) if not. The Wilcoxon/Kruskal–Wallis test was used for comparing the median d-CMP and the median d-GDA values between the groups of interest (vein resection vs. non-vein resection; stapler vs. harmonic shears). A logistic regression model was used to estimate the relationship between the continuous predictor (d-CMP, d-GDA) and the categorical response (vein resection vs. non-vein resection; stapler vs. harmonic shears). As an estimate of the effect size, the odds ratio (OR) was considered appropriate. The Receiver Operating Characteristic (ROC) curve was used to calculate the cut-off level of d-CMP for resecting the vein and of d-GDA for transecting the pancreas with a stapler or with harmonic shears. A *P* value < 0.05 was considered statistically significant (two-tailed). All statistical analyses were carried out with JMP^®^ 15.2.0 (SAS Institute Inc., Cary, NC, USA).

## Results

**Fig. 3:**
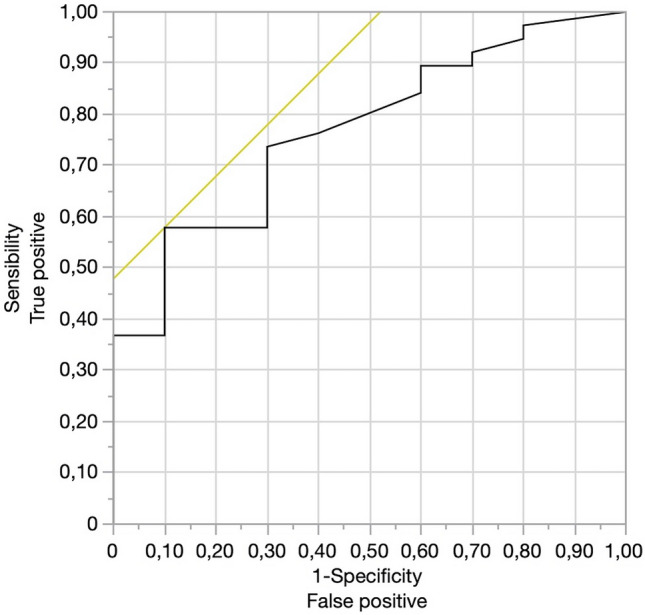
ROC curve for d-SMC. The cut-off value of d-SMC associated with vein resection was 17 mm (AUC 0.75)

**Fig. 4:**
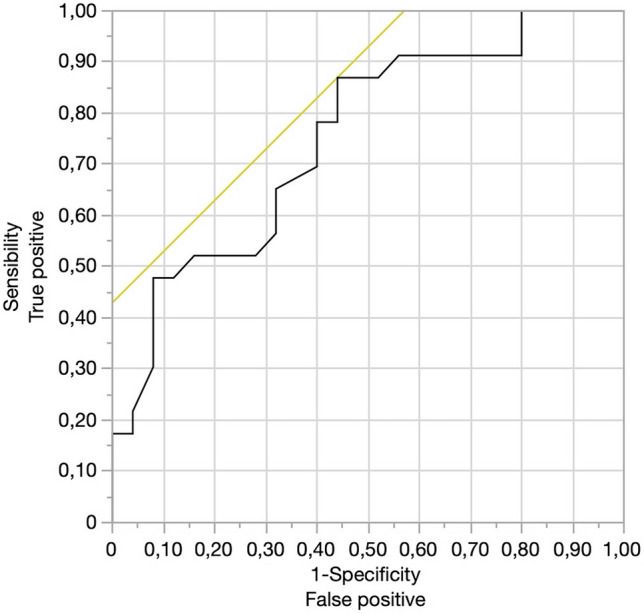
ROC curve for d-GDA. The cut-off value of d-GDA associated with the use of a stapler was 43 mm (AUC 0.75)

Between January 2011 and June 2022, 48 MI- RAMPS were performed. Baseline characteristics of the study population are summarized in Table 1. Three patients underwent conversion total pancreatectomy, following positive frozen histology at the neck margin. With a mean tumor size of 31.1 ± 14.7 mm, mean d- SMC was 21.5 ± 18.5 mm and mean d-GDA ± SD was 41.2 ± 23.2 mm.d-SMC was associated with the need for vein resection and reconstruction. In patients requiring a vein resection mean d-SMC was 10 mm (1.5–15.5) compared to 18 mm (10–37) in the group without vein resection (*p* = 0.01). The cut-off value of d-SMC associated with vein resection was 17 mm (AUC 0.75). (Fig. 3).d-GDA was associated with the possibility to use a stapler to divide the neck of the pancreas. Mean d-GDA was 46 mm (30–65) when the neck was divided using a stapler, and was 26 mm (19–39) mm when direct neck transection was required (*p* = 0.01). The cut-off value of d-GDA associated with the use of a stapler was 43 mm (AUC 0.75). (Fig. 4)

**Table 1:** Baseline characteristics

Patients, *n*	48
Mean Age ± SD, years	67.6 ± 8.6
Sex; male (%)	22 (45.8)
Mean BMI ± SD, Kg/m^2^	25.05 ± 4.3
Median ASA class [IQR]	3 [2, 3]
Neoadjuvant chemotherapy, *n* (%)	8 (16.6%)
Mean Ca19.9 ± SD, U/ml	245.7 ± 668,8
Mean tumor diameter at CT scan ± SD, mm	31.1 ± 14.7
Mean d-CMP ± SD, mm	21.5 ± 18.5
Mean d-GDA ± SD, mm	41.2 ± 23.2

*ASA* American society of anesthesiologists, *BMI* Body Mass Index, *IQR* interquartile range, *d-CMP* distance of the tumor from the left edge of spleno-mesenteric confluence, *d-GDA* The distance of the tumor from the gastroduodenal artery at its passage at the pancreatic head

Operative results are reported in Table 2. Laparoscopy and robotic assistance were used in 24 patients each. Conversion to open surgery occurred in 2 patients (4.1%), despite 10 patients required vein resection and 3 patients had a resection of the celiac trunk. There was one emergency conversion in a patient requiring resection of the celiac trunk. The neck of the pancreas was divided using a stapler in 25 patients (52.1%).

**Table 2:** Operative results

Robotic RAMPS, n (%)	24 (50.0%)
Laparoscopic RAMPS, *n* (%)	24 (50.0%)
Conversion, n (%)	2 (4.1%)
Elective	1 (2.0%)
Emergency	1 (2.0%)
Mean operative time ± SD, minutes	410.2 ± 157.6
Mean estimated blood loss ± SD, ml	631 ± 416.2
Intraoperative blood transfusions, *n* (%)	6 (12.5)
Stapled division of the neck of the pancreas, n (%)	25 (52.1%)
*SMC resection, n (%)	10 (20.8%)
Vein reconstruction	
Type 1	2 (20.0%)
Type 2	8 (80.0%)
Inferior mesenteric vein patch	4 (40.0%)
Middle colic vein patch	2 (20.0%)
Peritoneal patch	1 (10.0%)
Arterial patch from a deceased donor	1 (10.0%)
Celiac trunk resection, n (%)	3 (6.2%)

^*^SMC: splenomesenteric confluence

Postoperative results are reported in Table 3. Among the 10 patients who had a vein resection, two developed vein thrombosis. Both cases were successfully treated by intravenous heparin infusion. Clinically relevant postoperative pancreatic fistula developed in 12 (52.1%) after stapled division of the neck of the pancreas and 11 (47.9%) after sharp division of the neck of the pancreas (p = 0.99). The overall rate of clinically relevant postoperative pancreatic fistula was 47.9% (23/48), out of these grade C POPF was 0%.

**Table 3:** Post-operative and pathological results

Post-operative complications, *n* (%)	
Grade 0	12 (25.0%)
Grade 1	4 (8.3%)
Grade 2	24 (50.0%)
Grade 3	7 (14.6%)
Grade 4	1 (2.0%)
Grade 5	0
Severe post-operative complications, *n* (%)	8 (16.6%)
Comprehensive complication index, mean ± SD	21.9 ± 15.9
Length of hospital stay, days, mean ± SD	14.8 ± 8.5
Clinically relevant postoperative pancreatic fistula, *n* (%)	23 (47.9%)
Stapled division	12 (52.1%)
Sharp division	11 (47.9%)
Delayed gastric emptying, *n* (%)	9 (18.7%)
Grade A	7 (14.5%)
Grade B	1 (2.1%)
Grade C	1 (2.1%)
Postoperative pancreatic hemorrhage, *n* (%)	4 (8.3%)
Grade A	1 (2.1%)
Grade B	2 (4.2%)
Grade C	1(2.1%)
Chyle leak, *n* (%)	5 (10.4%)
Grade A	0 (0%)
Grade B	5 (10.4%)
Grade C	0 (0%)
Mean CCI	21.9 ± 15.9
30-day readimission, *n* (%)	3(6.2%)
Tumor histology, *n* (%)	
Pancreatic cancer	39 (81.2%)
Malignant Intraductal Mucinous Papillary Neoplasm	9 (18.8%)
T stage, *n* (%)	
T1	5 (10.4%)
T2	13 (27%)
T3	28 (58.3%)
T4	2 (4.1%)
*N* stage, *n* (%)	
N0	13 (27.1%)
N1	23 (47.9%)
N2	12 (25%)
R1, *n* (%)	28 (58.3)
Number of harvested nodes, mean ± SD	43 ± 16
Confirmed venous infiltration, *n* (%)	3 (30.0%)
Confirmed arterial infiltration, *n* (%)	2 (66.6%)

## Discussion

Not all distal pancreatectomies are the same. RAMPS aims to improve local tumor clearance but adds technical complexity over the conventional procedure [6, 7]. While several difficulty scores are available for MI-DP, none was specifically designed for MI-RAMPS. Dealing with pancreatic cancer, especially when the tumor is located in the body/neck of the pancreas, is associated with additional and specific challenges in the control of splenic vessels [6–9] and raises relevant oncologic issues. Tumor proximity to the SM-PV may require unplanned vascular resection in up to 60% of the patients [24], while positivity of frozen section histology at the neck margin may require conversion total pancreatectomy.

This study shows the practical relevance of two quite simple radiology measurements: d-SMC and d-GDA. When d-SMC is ≤ 17 mm, the need for vein resection should be taken into serious consideration. This piece of information has several practical implications. First, the surgeon can decide whether or not to proceed with a minimally invasive approach or could have a lower threshold for conversion to open surgery (that means also team preparation for that). Second, a plan for vascular reconstruction can be made (e.g. define the availability of vascular grafts). Third, these cases should not be used for training purposes, or assigned to beginners. Fourth, the use of robotic assistance should be preferred, since the robot could facilitate vascular reconstruction. Fifth, the anesthesia team can be informed about the anticipated increased difficulty of the procedure, including the need for vascular cross-clamping with its attendant implications on liver perfusion and systemic venous return (in a patient put under pneumoperitoneum and lying on an anti-Trendelenburg position).

When d-GDA is ≤ 43 mm, a stapler could not be used for the division of the neck of the pancreas. If the surgeon does not feel comfortable with alternative methods of pancreatic transection and closure, he or she should consider to proceed with an open procedure. Try to forcibly insert the stapler in a narrow and deep retropancreatic tunnel should be avoided. In addition, when the stapler is fired very close to the tumor, a few millimeters of pancreatic tissue are lost within the staple lines and are not available for frozen section histology of the neck margin.

Despite this issue not specifically addressed in this study, when d-SMC is ≤ 17 mm and d-GDA is ≤ 43 mm, MI-RAMPS promises to require several variations from the “standard” technique reported in textbooks. Depending also on the type of splenic artery bifurcation [25], technical difficulty in these patients could be truly high.

The importance of tumor proximity to major vessels in minimally invasive distal pancreatectomy was already reported in several difficulty scores [6–9]. The rate of RAMPS in these studies varied between 6 and 38% [6, 7], while only one study reported about the need for vein resection (1%) [6]. In none of these studies, vein resection was reported among the reasons for conversion to open surgery. Conversion rates ranged between 0 [7] and 19% [6]. Need for vein resection is generally disregarded as a reason for conversion in MI-DP [26], possibly underscoring the use of stringent selection criteria that exclude these patients from a minimally invasive approach. In open distal pancreatectomy, vein resection is required in approximately 10% of the patients [27].

In this study, 10 patients (21%) required a vein resection and 3 patients required also a resection of the celiac trunk (6%). These figures could either reflect the fact that was specifically reported on RAMPS for pancreatic cancer and malignant intraductal papillary mucinous neoplasms, or our overall experience in pancreatectomy with vascular resections, that currently exceeds 600 procedures [28]. On practical grounds, this relatively high prevalence of associated vascular procedures in our series offered to opportunity to define a cut-off value of d-SMC that anticipates the need for vein resection in MI-RAMPS.

Few other results from this study deserve a specific comment. In particular, it is reported a seemingly high rate of R1 resections (58%). Considering the high number of examined lymph nodes (mean: 43 ± 16) it is possible that this rate spells more for accurate pathology than for “non-radical” surgery [29]. In a recent study on robotic pancreatoduodenectomy with radical en-bloc clearance of the triangle space (so-called mesopancreas), our group also reported quite high R1 rates (44%) despite lymph node yields three-fold higher than current pathology standards [30]. These data reinforce the concept that “most pancreatic cancer resections are R1 resections” [31] and suggest that data on low R1 rates following pancreatectomy for pancreatic cancer should be carefully and critically evaluated.

This study has some limitations. First, the retrospective study design carries the risk of selection bias such as having operated through a MI access ro patients who should have received an open procedure, and vice-versa. Second, rates and outcomes of vascular resections in MI-RAMPS may correspond to a center-specific experience influenced by hundreds of open procedures and tenths of minimally invasive operations. Therefore, the generalizability of our results remains to be established. Third, not all centers systematically pursue MI-RAMPS for pancreatic cancer. Performing a potentially less radical procedure could improve postoperative outcomes.

In conclusion, two simple radiology measurements on preoperative CT scan (d-SMC and d-GDA) have important practical implications on MI-RAMPS performed for pancreatic cancer and malignant intraductal papillary mucinous neoplasms.

## Data Availability

The datasets generated during the current study are available from the corresponding author on reasonable request.
